# Association between dependency on community resources and social support among elderly people living in rural areas in China: a cross-sectional study

**DOI:** 10.1186/s12877-022-03247-5

**Published:** 2022-07-16

**Authors:** Ayizuhere Aierken, XiWen Ding, YiYang Pan, Yuan Chen, Ying Li

**Affiliations:** Department of Social Medicine, School of Public Health, Zhejiang University, 866 Yu-hang-tang Road, Hangzhou, 310058 Zhejiang China

**Keywords:** Dependency, Community resources, Rural areas, Social support, Elderly people, Self-efficacy

## Abstract

**Background:**

The prevalence of dependency personality disorder (DPD) is high among elderly people living in rural areas. This study aims to explore the association between dependency on community resources and social support among elderly individuals living in rural areas.

**Methods:**

A cross-sectional study was conducted in 26 locations in China. A total of 1160 participants aged ≥ 60 years were selected using a complex multistage sampling design. All data were obtained using questionnaires via face-to-face interviews. DPD was measured using the Minnesota Multiphasic Personality Inventory-II in the standardized Chinese version. Self-efficacy was assessed using the Chinese version of the General Self-Efficacy Scale. Social support was measured using the Chinese version of the questionnaires of the Older American Resources and Services scale. Community services and resources comprised 44 items. The association between DPD and levels of social support and self-efficacy was evaluated using a logistic regression model. The association between social support and self-efficacy was assessed using analysis of covariance.

**Results:**

Univariate analysis results showed that elderly people living in rural areas had higher DPD scores and lower levels of self-efficacy compared with those living in urban areas (*P* < 0.001). Logistic regression analysis showed that DPD was positively associated with the received frequencies of community health service, contracted family doctor services, and regular lectures on health knowledge among the elderly people with odd ratios of 1.58 (*P* < 0.001), 2.03 (*P* = 0.013), and 2.67 (*P* = 0.005), respectively. Logistic regression analysis showed significant interaction between social support and self-efficacy effect on DPD was found in the additive model (*P* < 0.001).

**Conclusion:**

DPD was associated mainly with the community resources among elderly people living in rural areas. Social support and self-efficacy were commonly associated with DPD through a synergistic effect. These results suggest that DPD among elderly people may be reduced through effective social support to directly and indirectly promote the elderly’s use of community resources and improve their self-efficacy.

**Supplementary Information:**

The online version contains supplementary material available at 10.1186/s12877-022-03247-5.

## Background

The way the world population is aging has resulted in the rapid increase in the number of elderly people with declining physical function and those having chronic diseases. Additionally, since the prevalence of dependency personality disorder (DPD) increases with age, especially in the elderly people with lower social capital, it is rapidly becoming an urgent, enormous challenge for individuals and social service systems because it requires immediate solutions [1–3]. However, the association between DPD and health or provision-of-support services is complicated [4].

In case of dependency personality disorder, individuals become highly reliant on others to meet their emotional and physical needs, resulting in the gradual loss of autonomy [5]. An attachment to different dependency objects can lead to nursing dependency, substance dependency, or sleep dependency. Epidemiological studies have demonstrated that DPD can also increase the consequences of chronic diseases, and all-cause mortality [6, 7]. Moreover, DPD is associated with the overuse of healthcare resources, and can lead to premature functional dependency and disability [8].

The etiologic mechanism of DPD is still unclear, but the consequences of DPD and poor health are intimately associated with social environmental factors, especially in elderly people [9]. The prevalence of DPD is high among elderly people in traditional areas, such as the rural regions of China [10]. For a long time, families were considered as their major healthcare resources [11]. However, with the rapid development of society and economy, young people move to urban areas and gradually forget traditional culture or lose the ability to take care of elderly people. Hence, family members cannot be considered as the major healthcare resource for elderly people living in rural areas. In addition, these elderly people would rather live at home than go to a nursing home even when their health condition has deteriorated to the point where they need urgent and emergency care. With aging, elderly people develop complex and increased needs that require sustained input from community health service systems to support independent living. Socially disadvantaged elderly people and those without family to provide unpaid care and sufficient income might prematurely fall into DPD [12]. For elderly people living in rural areas, the local resource is particularly important for their health; however, their community resources are inadequate [13]. A prospective study reported that the availability of social support is an important factor for possible assistance with DPD [14]. Moreover, resource inequality is a barrier in providing the necessary support for independence [15, 16]. The effect of social support on the health outcome of elderly people has long been a topic of concern. Although social support has direct and indirect effects on mental health and DPD, the way of providing social support and the potential mechanism for social support as a mediator related to individual’s mental health remain clear [17, 18].

Previous studies on DPD mostly focused on negative consequences; however, recent findings suggest that dependency on health service resources may be considered as a positive condition for individuals with DPD because it can meet the individual’s demand for safe [19, 20]. The state of dependency is dynamic and ever-changing through use of community health service resources. Dependency on community health service resources has been considered a helpful intermediate process from dependency to autonomy [21]. Therefore, it is not an entirely negative event.

Resource dependency theory has escribed the relationship between a resource and resource users [22, 23]. Resource users are highly dependent upon a resource due to they can obtain social and health benefits. To understand why and how individuals are dependent on a resource may provide insights into the condition of resource users and meeting the needs of resource. The objective of this study is to determine the DPD-associated-with-community resources among elderly people living in rural areas the theory of resource dependency and explore further suitable methods to reduce and delay the DPD in elderly people, and to alleviate the problem of community resource shortage in rural areas. In addition, the theory that social support promotes behavior change was verified using the health belief model. The health belief model is one of the first theories of health behavior and remains one of the most widely recognized theories in behavioral science and public health [24]. This model hypothesizes people’s beliefs as to whether individual is at high risk for disease and their perspectives of the benefits of taking necessary action to avoid it potentially affect their readiness to act. The recent additions of self-efficacy are the core constructs of the health belief model. A previous study used the health belief model combined with health counseling from local public health authorities to improve the hospital visit rate [25]. The results showed that the frequency of hospital visits in the intervention group increased, and the cumulative incidence of lifestyle-related diseases decreased after the physical examination. We hypothesized that if strategies for improving community resources or behavior changes can be provided based on health belief theory, then it is possible for elderly people to live independently in their familiar and dependent communities for a long time.

## Methods

### Subjects

This study was included in the “Accessibility Evaluation of Health-Related Resources for the Elderly” project. A population-based cross-sectional study was conducted to assess the health and health-related accessibility resources and services among elderly people in rural and urban areas. A total of 1160 community residents aged ≥ 60 years were selected using a multistage sampling design. Sampling was conducted in 26 locations (11urban/15rural) in four provinces (Zhejiang, Heilongjiang, Xinjiang, and Sichuan) in China from July 2019 to August 2021. Inclusion criteria were as follows: permanent residents of aged 60 years and above, able to answer and understand the study questionnaire, and agreed to participate in the study. Exclusion criteria were as follows: having severe physical and mental disorders and hearing impairment that leads to communication difficulties and unable to complete the questionnaire. All participants provided written informed consent before joining. The study was approved by the institutional review board at the School of Medicine, Zhejiang University (No: ZGL201909-10) and performed in accordance with the Declaration of Helsinki.

### Data collection

All data from the program were collected by face-to-face interviews using structured questionnaires. The questionnaire included nine parts and comprised 428 items and other two instruments for measuring cognitive function that uses separate answer sheets. The main contents comprised categories, such as demographics, lifestyle and behavioral habits, self-reported chronic diseases and general health status, environmental and community health service resources, socioeconomic resources, psychological and cognitive assessment, and activities of daily living assessment. The participants were informed in advance of the time required for the investigation. The interview lasted approximately 45–60 min for the majority of the participants.

#### Characteristic variables

The general characteristics of the participants included age, gender, marital status, income, living arrangement, education level, self-reported health status, and daily behavior and habits. The daily behavior and habits included about smoking, drinking, physical activity, and food intake.

#### Community resources

Community resources comprised 44 items [26], and the Cronbach’s alpha coefficient was 0.89 for total scale. The main questions were as follows: “In the past six months, how many times have you received treatment or a general practitioner’s onsite service from community health service organizations”? The answers were divided into four levels of “ < 2 times,” “2–3 times,” “4–6 times,” and “ > 6 times.” “Do you think someone must examine and assess your overall health status?” “Does your community have a family doctor contract service?” “Does your community conduct regular lectures on health knowledge and use electronic health records?” “Does your community have a counseling service?” “Is there an elder university, cultural activity center or geriatric ward in your community” “Does your community have an emergency call or survival monitoring service?”. If the respondents replied “yes” to these questions, then the answer was coded as “1”; otherwise, it was coded as “0”. Many other questions were also asked as follow: “Does your community have a day care service?” “Does your community have a transfer service?” “Does your community hold regular outdoor activities and reemployment guidance?” and “Does your community have medication guidance and care services for chronic patients.

### Measurements

#### Dependency personality disorder

The Chinese version of the standardized Minnesota Multiphasic Personality Inventory-II was used to assess the DPD among the elderly. The DPD scale comprised 57 items, and the test–retest reliability were 0.67 in man and 0.81 in women [27]. The raw score was calculated in accordance with the instruction manual and converted into a standardized T-score. DPD was defined as a standardized T-score greater than or equal to 60 points.

#### Self-efficacy

The Chinese version of the General Self-Efficacy Scale (GSES) was used to assess self-efficacy, and had a Cronbach’s alpha of 0.89 [28]. The GSES consisted of 10 items with a four-point rating scale for a total of 10–40 points, with high scores indicating high levels of self-efficacy.

#### Social support

Social support was assessed using the Chinese version of the questionnaires of the Older American Resources and Services (OARS) social resource scale. The ratings were summed up to yield a total score, and Cronbach’s alpha coefficients ranged from 0.61 to 0.83 [29]. A high level of social support was defined as an OARS score greater than or equal to 11 points.

#### Depressive symptom

Depressive symptoms were assessed using the 15-item Geriatric Depression Scale (GDS-15). The optimal cut-off point to identify depressive symptoms was 5 points; 0–4 points indicated no depressive symptoms, 5–9 points indicated mild depressive symptoms, and ≥ 10 points indicated moderate to severe depressive symptoms [30].

#### Personality characteristics

Participants’ personality characteristics were measured using the Eysenck Personality Questionnaire (EPQ), which consists of 88 items and is designed to assess personality dimensions of extraversion, neuroticism, and psychoticism. High scores are highly indicative of an Eysenck personality [31].

### Statistical analysis

The general characteristics of participants were described using frequencies and percentages. Chi-square tests were used to compare the characteristics of community resources among the participants living in urban and rural areas.

The association between DPD and community resources in urban and rural areas was evaluated using two separate logistic regression models. All models were adjusted for gender, age, educational level, marital status, individual income, smoking status, alcohol use, levels of physical activity, chronic disease status, EPQ score, and community resources. Logistic regression models were also used to assess the association between DPD and social support or self-efficacy and tested multiplicative and additive interaction. In the additive interaction model, social support and self-efficacy scores were treated as a binary variable. If the participant’s self-efficacy score is greater than 25 points, then it will be regarded as a binary dependent variable expressed by “1”; otherwise, the grade is “0”. A binary variable of social support level was also created using the same method, and three new dummy variables were generated using the two binary variables. All independent variables were added to the logistic regression model through a stepwise method.

Analysis of covariance (ANCOVA) was performed to evaluate the association between the level of self-efficacy and social support score. Normality and variance homogeneity tests were conducted prior to the ANCOVA. The means and standard errors of social support scores were calculated using the levels of self-efficacy. The three categories were compared using the F test, and a linear trend was evaluated using a general linear model.

All analyses were performed at a significance level of *P* < 0.05 for two-sided tests by using SAS for Windows (version 9.4).

## Results

The general characteristics of the study participants are shown in Table 1. Among the participants, 717 (63.3%) live in rural areas and 415 (36.7%) live in urban areas with an average age of 68 years. Approximately 24.7% and 4.6% of the participants living in rural and urban areas, respectively, reported that they completed only 6 years or less of education. More than 72.9% of the participants living in rural areas reported that they have a low level of income, and only 10.4% of the participants living in urban areas reported that they earn less than 2,000 yuan per month. Elderly people living in rural areas had higher DPD scores and lower levels of self-efficacy compared with those living in urban areas (*P* < 0.001). The mean GDS-15 score were 3.7 points for the participants living in rural areas and 2.2 points for those living in urban areas.

**Table 1:** Characteristics of participants in the study

Variable categories	Rural (*N* = 717)		Urban (*N* = 415)	
	n	%	n	%
Age (yr)				
60–69	430	60.0	269	64.8
70–79	232	32.4	132	31.8
≥ 80	55	7.6	14	3.4
Gender				
Male	326	45.5	129	31.1
Female	391	54.5	286	68.9
Marital status				
Married	566	78.4	367	85.7
Non-married	156	21.6	61	14.3
Education (yr)				
0–6	177	24.7	19	4.6
7–9	334	46.6	112	27.0
10–12	142	19.5	135	32.5
13 +	66	9.2	149	35.9
Individual income				
¥0 to 1,999	523	72.9	43	10.4
¥2,000 to 3,999	139	19.4	257	61.9
¥4,000 to 5,999	48	6.7	73	17.6
¥6,000 and Over	7	1.0	42	10.1
Smoking status				
Yes	162	22.6	49	11.8
No	555	77.4	366	88.2
Alcohol use				
Yes	170	23.7	128	30.8
No	547	76.3	287	69.2
Physical activity				
Yes	224	31.2	114	27.5
No	493	68.8	130	72.5
Chronic disease status				
Yes	475	66.2	305	73.5
No	242	33.8	110	26.5
Measured Variables (Mean, SD)				
Dependency scores	44.2	12.4	40.1	12.0
Self-efficacy scores	24.9	6.7	25.6	6.0
GDS-15 scores	3.7	3.0	2.2	2.1
EPQ scores	22.2	4.2	23.2	3.3

The main difference in community resources among the participants living in rural and urban areas is shown in Table 2. Univariate analysis results indicated that the received frequencies of community health service in the past 6 months were significantly lower among the participants living in rural areas compared with those among the elderly people living in urban areas (*P* < 0.001). The participants living in rural areas had a significantly lower utilization rate of community service and resources, such as family doctor contract service, community counseling service, emergency call system, elder university, and community cultural activity center, than those living in urban areas. The participants living in rural areas had a higher need for health assessment and a lower level of social support than those living in urban areas. All *p*-values were less than 0.05.

**Table 2:** The description of community services and resources among participants in rural and urban areas

Variable	Rural		Urban		*P* value
	n	%	n	%	
Received community health services (times)					
< 2	497	69.3	199	47.9	
2–3	132	18.4	86	20.7	
4–6	53	7.4	70	16.9	
> 6	35	4.9	60	14.5	< 0.001
The need for health assessment					
Yes	651	90.8	355	85.5	
No	66	9.2	60	14.5	0.006
Family doctor contract service					
Yes	319	44.5	326	78.5	
No	398	55.5	89	21.5	< 0.001
Community counseling service					
Yes	50	7.0	49	11.8	
No	667	93.0	366	88.2	0.005
Elder university					
Yes	101	14.1	181	43.6	
No	616	85.9	234	56.4	< 0.001
Community cultural activity center					
Yes	258	36.0	233	56.1	
No	459	64.0	182	43.9	< 0.001
Emergency call or monitoring system					
Yes	97	13.5	149	35.9	
No	620	86.5	266	64.1	< 0.001
The level of social support (scores)					
≤ 10	332	46.3	140	33.7	
> 10	385	53.7	275	66.3	< 0.001

The odds ratios (ORs) of DPD association with community services and resources by logistic regression analysis are shown in Table 3. Model 1 showed that among the participants living in rural areas, the DPD was positively associated with received frequencies of community health service, contracted family doctor services, and regular lectures on health knowledge with ORs of 1.58 (95% CI, 1.21–2.07, *P* < 0.001), 2.03 (95% CI, 1.16–3.56, *P* = 0.013), and 2.67 (95% CI, 1.34–5.32, *P* = 0.005), respectively. The community cultural activity center with an OR of 0.35 (95% CI, 0.18–0.70, *P* = 0.003) and the need for health assessment with an OR of 0.25 (95% CI, 0.10–0.61, *P* = 0.002) were negatively associated with DPD. Model 2 showed that among the participants living in urban areas, DPD had a positive association with community geriatric ward and utilization of electronic health records with ORs of 2.88 (95% CI, 1.28–6.48, *P* = 0.011) and 5.32 (95% CI, 1.80–15.73, *P* = 0.002), respectively, and had a negative association with elder university with an OR of 0.39 (95% CI, 0.18–0.84, *P* = 0.016).

**Table 3:** The odds ratios of dependency association with community services and resources by logistic regression analysis

Variables	Multivariable adjusted			*P* value
	Odd Ratios	95% CI		
Rural (model 1)				
Received community health services (times)	1.58	1.21	2.07	< 0.001
Regular lectures on health knowledge (n/y)	2.67	1.34	5.32	0.005
Community cultural activity center (n/y)	0.35	0.18	0.70	0.003
The need for health assessment (n/y)	0.25	0.10	0.61	0.002
Family doctor contract service (n/y)	2.03	1.16	3.56	0.013
Community geriatric ward (n/y)	0.30	0.07	1.24	0.097
Elder university (n/y)	2.44	0.56	1.62	0.237
Utilization of electronic health records (n/y)	1.04	0.28	3.78	0.957
Urban (model 2)				
Received community health services (times)	1.03	0.68	1.58	0.876
Regular lectures on health knowledge (n/y)	0.40	0.12	1.37	0.147
Community cultural activity center (n/y)	0.73	0.25	2.12	0.558
The need for health assessment (n/y)	0.46	0.11	1.95	0.289
Family doctor contract service (n/y)	1.96	0.46	8.39	0.364
Community geriatric ward (n/y)	2.88	1.28	6.48	0.011
Elder university (n/y)	0.39	0.18	0.84	0.016
Utilization of electronic health records (n/y)	5.32	1.80	15.73	0.002

The results of the association between DPD and social support and self-efficacy are shown in Table 4. High levels of social support and self-efficacy were associated with low DPD scores with ORs of 0.75 (95% CI, 0.65–0.86, < 0.001) and 0.92 (95% CI, 0.86–0.98, *P* = 0.016), respectively. The levels of social support and self-efficacy had no significant effect on DPD scores in the multiplicative model but had a significant interaction in the additive model (*P* < 0.001). In the additive model with dummy variables, after the interaction items were added to the logistic regression model, the levels of social support had an OR of 0.57 (95% CI, 0.46–0.68, *P* < 0.001) and self-efficacy had an OR of 0.70 (95% CI, 0.58–0.82, *P* < 0.001). The interaction items for the levels of social support and self-efficacy had an OR of 0.34 (95% CI, 0.19–0.48, *P* = *P* < 0.001).

**Table 4:** The association between dependency and social support or self-efficacy by logistic regression analysis

Variables	Multivariable adjusted			*P* value
	Odd Ratios	95%	CI	
Model 1				
The levels of social support (points)	0.75	0.65	0.86	< 0.001
The levels of self-efficacy (points)	0.92	0.86	0.98	0.016
Education levels (low/high)	0.62	0.59	0.98	0.043
EPQ scores (points)	1.25	1.13	1.39	< 0.001
Gender (m/f)	3.74	1.60	8.78	0.002
LSS*LSE (Multiplicative interaction)	0.21	0.01	0.46	0.064
Model 2				
The levels of social support (low/high)	0.57	0.46	0.68	< 0.001
The levels of self-efficacy (points)	0.70	0.58	0.82	< 0.001
Education levels (low/high)	0.93	0.54	1.63	0.592
EPQ scores (points)	1.23	1.17	1.30	< 0.001
Gender (m/f)	0.94	0.78	1.20	0.821
LSS + LSE (Additive interaction)	0.34	0.19	0.48	< 0.001

*LSS* Levels of social support

*LSE* Levels of self-efficacy

*Multiplicative interaction symbol

**Fig. 1:**
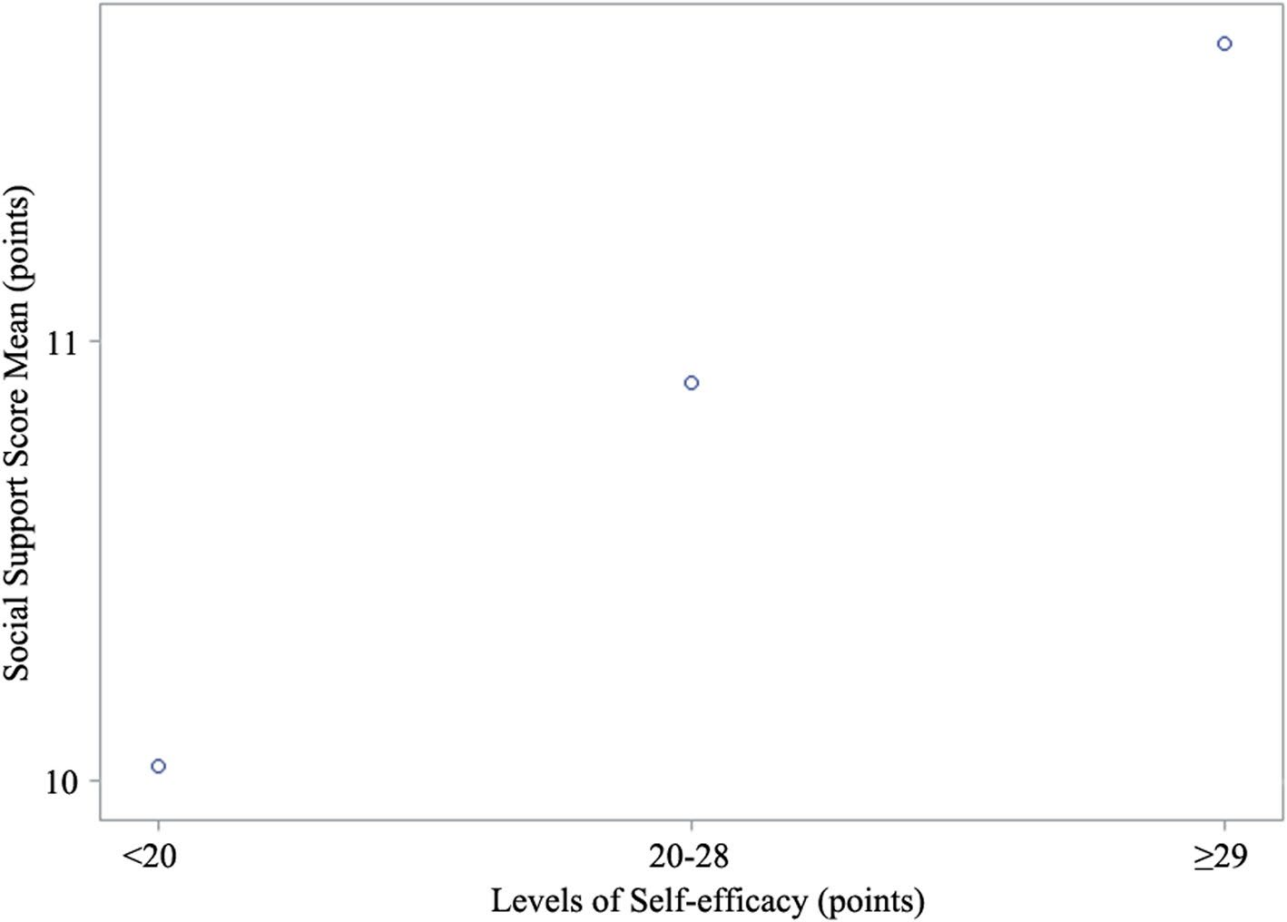
The means and standard errors of social support scores for the levels of self-efficacy

As illustrated in Table 5 and Fig. 1, the mean ANCOVA scores of social support for the different levels of self-efficacy were 9.92, 10.88, and 11.59 points in the three level groups “ < 20,” “20–28,” and “ ≥ 29”, respectively, resulting in a significant linear trend (*P* < 0.001).

**Table 5:** The levels of self-efficacy association with social support by ANCOVA

	Social support scores (points)			
	Means	Standard error	*P* value	*P* for trend
The levels of self-efficacy				
< 20 (points)	9.92	0.17		
20–28 (points)	10.88	0.10	< 0.001	
≥ 29 (points)	11.59	0.13	< 0.001	< 0.001

## Discussion

This study found that elderly people living in rural areas had a significantly higher level of DPD and lower level of social support and self-efficacy compared with those living in urban areas. The rural community-dwelling elderly people had few community resources and utilization of health service, and their DPD was positively associated with the frequency of received community health service, contracted family doctor services, and regular lectures on health knowledge. Further study showed that social support and self-efficacy had an additive interaction effect on DPD.

Previous studies found that the prevalence of psychiatric disorders, such as alcohol and tobacco dependency, is higher in rural areas than in urban areas. In addition, tobacco cultivating status was confirmed to be an important risk factor of tobacco dependency [32–34]. However, studies have suggested that the high prevalence of DPD among the elderly people living in rural areas is mainly due to social and economic factors [35]. The focus of studies on DPD has also changed with the continuous development of society and our understanding of the association between health and society. A previous study showed that the support of dependent elderly is conditioned upon the availability of the family, particularly of children. However, determining whether the elderly will have children capable of providing for them is difficult [36]. With the development of the economy and society over the past 30 years, the number of left-behind elderly people has increased rapidly in rural China and other countries [37]. These elderly people have increased DPD risk with regard to physical, mental, social, and economic areas; however, a study showed that only an extremely small proportion of elderly people are physically dependent [38]. Although economic status is mainly associated with the DPD of elderly people, special government funding has not solved this complex problem [39]. Another study suggested that an evidence-based approach is necessary to understand the association between community resources and DPD among elderly people, but only a few investigations have conducted comprehensive analyses of elderly people living in rural areas [40, 41].

In the present study, elderly people living in rural areas were also characterized by a low level of social support and few health-support services and resources. The elderly people living in rural areas were significantly dependent on received community health services, contracted family doctor services, and regular lectures on health knowledge. This finding is similar to previous results stating that DPD may lead to the overuse of healthcare resources [42]. However, in contrast with the negative results, recent studies suggested that dependency on community resources may be regarded as a positive condition for individuals based on the theory of resource dependency. Dependency might be a necessary and helpful temporary phase toward autonomy [43]. On the basis of this theory, the present results suggested that elderly people living in rural areas with a high level of DPD have a great demand for community resources, such as general community health services, contracted family doctor services, and regular lectures on health knowledge. If they can be provided with the appropriate services and social support, then the elderly people with a high level of DPD will gradually move toward autonomy.

Another result also confirmed the conclusion from the present study. Compared with those whose health status has not been evaluated by doctors, the elderly people whose overall health has been examined or thoroughly assessed by a doctor in the past 6 months believe it is necessary to check or evaluate their health status and have a low level of DPD. DPD is associated with health promotion behavior. These people will quickly ask for the help of doctors after a physical examination, thus increasing the degree of cooperation and compliance during treatment. The theory of health belief model has also been confirmed in the present study. In this study, the association between self-efficacy and DPD was also explored. High levels of self-efficacy and social support were associated with low DPD scores, and an additive interaction was observed between self-efficacy and social support. The self-efficacy and interaction items were kept statistically significant in the model, and the three dummy variables representing self-efficacy, social support, and interaction item were added to the logistic regression model. The results suggested that self-efficacy may be directly or indirectly associated with DPD, and self-efficacy and social support are commonly associated with a low level of DPD through a synergistic effect.

Social support is affected by culture, socioeconomic factors, and policies and impacts health through health behavioral pathways or psychological pathways [44]. Social support may affect physical, cognitive, and mental health outcomes and the overall social function and engagement of elderly people [45]. Self-efficacy is an important mediator between social support and health. Integrated interventions of promoting perceived social support may be effective in enhancing self-efficacy [46]. Elderly people face life adversities, poor social support, and limited access to health services, which may affect their health self-efficacy [47]. Self-efficacy is the faith that an individual can successfully execute behaviors to achieve desired aims and improves the ability to change substance use behaviors [48]. Some studies showed a correlation between self-efficacy and individuals with alcohol addiction. Self-efficacy has been proposed as a key predictor of alcohol treatment outcomes and a potential mechanism of success in achieving abstinence following alcohol treatment. In addition, self-efficacy might contribute to cognitive and behavioral changes. Although these previous studies mainly explored the association among social support, self-efficacy, and DPD and the underlying mechanisms, their results provided a strong support for our findings. Self-efficacy is a potentially important factor for mediating the use of community services to reduce dependency based on the health belief model. The present study argued that if elderly people living in rural areas are provided with adequate community health services, contracted family doctor services, and regular lectures on health knowledge, then they will quickly ask for the help of doctors and develop increased self-efficacy to take beneficial action to promote their autonomy and avoid DPD. On the one hand, additional social support can directly reduce the DPD of elderly people. On the other hand, social support can also reduce the risk of DPD by increasing self-efficacy among elderly people.

This study has one important limitation that must be addressed: its cross-sectional design. The associations between DPD and community health services and resources from logistic regression analysis were observed, but the causality could not be discussed further. The results showed that low scores of DPD were associated with a high level of social support and self-efficacy, which initially proved the assumption of this study that providing additional social support can prompt individuals to move from dependence on community resources to autonomy. In addition, the association was clarified using the theory of health belief model. However, only the interaction between social support and self-efficacy effect on DPD was statistically significant. Therefore, the complex and changing trends of behavior factors and the risk of DPD over time cannot be evaluated.

## Conclusions

This study showed that DPD was positively associated with the frequency of received community health services, contracted family doctor services, and regular lectures on health knowledge among elderly people living in rural areas. The high scores of social support were associated with a high level of self-efficacy, and both are important impact factors associated with the low level of DPD. A significant interaction was also observed among these factors. This study suggested that DPD is associated with an increase in the need and use for community resources. Providing additional social support can directly reduce the level of dependency on community resources. In addition, individuals can gradually move from dependency to autonomy by increasing their level of self-efficacy. In conclusion, effective social support can be an effective strategy for increasing the self-efficacy of elderly people and improving the utilization of rural community resources. An enhanced self-efficacy would allow these people to take beneficial action to promote their autonomy and avoid DPD.

## Supplementary Information

**Additional file 1.** Accessibility evaluation of health-related resources for the elderly.

## Data Availability

The datasets generated and analysed during the current study are not publicly available due to ensure the privacy of participants but are available from the corresponding author on reasonable request.

## Abbreviations

GSES: General Self-Efficacy Scale
OARS: Older American Resources and Services
EPQ: Eysenck Personality Questionnaire
ANCOVA: Analysis of covariance
GDS-15: Geriatric Depression Scale
ORs: Odds ratios
