# Supplementary material for: Association between dependency on community resources and social support among elderly people living in rural areas in China: a cross-sectional study
Source: BMC Geriatr. 2022 Jul 16;22:589. doi: 10.1186/s12877-022-03247-5 (PMC9288718; doi:10.1186/s12877-022-03247-5)
Supplement: Supplementary file 1 — Additional file 1. Accessibility evaluation of health-related resources for the elderly. [file 12877_2022_3247_MOESM1_ESM.doc]

**Accessibility Evaluation of Health-Related Resources for the Elderly**

**JUL 2019**

**School of Public Health, Zhejiang University**

**CONTENTS**

A DEMOGRAPHIC BACKGROUNDS

B CONCEPTION OF PROVIDING FOR THE AGED

C ASSESSMENT OF DEPENDENT PERSONALITY

D SOCIAL RESOURCE CONDITIONS

E MEDICAL SERVIC CONDITIONS

F FACILITIES AND SERVICES AVAILABLE IN COMMUNITY

G THE MEASUREMENT OF SELF-EFFICACY

H ASSESSMENT OF COGNITION AND PHYSICAL FUNCTION

I ASSESSMENT OF GERIATRIC DEPREESION

J MENTAL HEALTH

K GENETIC AND ENVIRONMENTAL HEALTH

L THE COMPREHENSIVE ASSESSMENT OF PERSONALITY

**A DEMOGRAPHIC BACKGROUNDS**

**A1** Interviewer record interviewee’s gender.

1.Male

2.Female

**A2** When were you born? _____year_____month_____day

What is your age? _____years old

**A3** What’s your nationality?

1.Han *Skip AA3*

2.Ethnic Minorities **AA3**：_____

**A4** What’s your current permanent address?

_____city_____county/district_____street/town

How long have you lived here? _____years

**A5** What’s your highest level of education?

1.No formal education (illiterate)

2.Elementary school or below

3.Middle school

4.High school or vocational school

5.Two-/Three-Year College/Associate degree or Four-Year College/Bachelor’s degree

6.Master’s degree and above

**A6** What’s your marital status?

1.Never married

2.Married

3.Divorced

4.Widowed

5.Remarried

**A7** Do/Did you smoke?

1.No *Skip to A8*

2.Sometimes

3.Often

4.Quit *Skip to A73*

**A71** What kind of cigarette do you mainly smoke?

1.＜10 Yuan per pack

2.10-19 Yuan per pack

3.20-29 Yuan per pack

4.30-39 Yuan per pack

5.≥40 Yuan per pack

**A72** In one day about how many cigarettes do you consume?

1.＜2 cigarettes

2.2-3cigarettes

3.4-5 cigarettes

4.≥6 cigarettes

**A73** At what age did you start to smoke? ____years old

**AA73** At what age did you quit smoking? ____years old

**A8** Do/Did you drink?

1.No *Skip to A9*

2.Sometimes

3.Often

4.Quit *Skip to A84*

**A81** What type of alcoholic beverages did you drink?

1.Beer

2.Liquor

3.Rice wine

4.Wine

5.Mixed wine

**A82** How often do you drink?

1.＜twice a week

2.2-3 times a week

3.4-5 times a week

4.≥6 times a week

**A83** How many liang of liquor did you drink?(1 liang = 50cc/50ml) ____liang

**A84** At what age did you start to drink? ____years old

**AA84** At what age did you quit drinking? ____years old

**A9** Do you regularly participate in sports such as hiking, jogging, playing ball, cycling or swimming, etc.

1.Yes **AA9** What sports do you mainly participate in：____

2.No *Skip to A10*

**A91** In the past month, about how many times have you participated in such sports?

____times/month

**A92** How long did you exercise each time?____minutes each time

**A93** Did you sweat or slightly sweat every time you exercise?

1.Yes

2.No

**A10** During the past month, how many hours of actual sleep did you get at night (average hours for one night)? (This may be shorter than the number of hours you spend in bed.) ____hours

**A101** What time do you usually go to bed? ____a.m./p.m.

**AA101** What time do you usually get up? ____a.m./p.m.

**A102** How long does it take you to really fall asleep? ____minutes

**A103** During the past month, how long did you take a nap after lunch? ____minutes

**A11** Do you eat regularly every day?

1.Very regular

2.Regular

3.Not very regular

4.Irregular

**A111** How many meals do you normally eat every day? ____meals

**AA111** How often do you eat snacks or fruits every day? ____times

**A12** Do you have the habit of drinking tea?(more than once a week)

1.Yes

2.No *Skip to A13*

**A121** What kind of tea do you usually drink?

1.Green tea

2.White tea

3.Yellow tea

4.Oolong tea

5.Dark tea

6.Black tea

**A122**  How often do you drink tea?

1.＞3 times a day

2.2-3 times a day

3.once a day

4.4-6 times a week

5.1-3 times a week

**A13**  Do you have a mobile phone?

1.Yes

2.No *Skip to A14*

**A131** What type of phone are you using?

1.Ordinary mobile phone

2.Mobile phone for the elderly

**A132** Does your mobile phone have Internet access?

1.Yes

2.No

**A133** What functions do you usually use?

1.Make a call

2.Send message

3.WeChat

4.Health management platform

5.Shopping platform

6.Watch news/entertainment

7.Other:____

**A14**  Are you currently receiving pension?

1.Yes

2.No

**A15**  What is your main source of income?

1.Wage

2.Pension

3.Provided by family members

4.Government subsidy

5.Other:____

**A16** What is your income?

1.＜2000 Yuan

2.2000-3999 Yuan

3.4000-5999 Yuan

4.6000-7999 Yuan

5.≥8000 Yuan

**A17** Do you think your income is sufficient to meet your basic needs?

1.Yes

2.No

**A18** Do you have any spare money to buy some small luxury goods?

1.Yes

2.No

**A19** If you want, can you eat eggs, meat and fish every day?

1.Yes

2.No

**A20** Is your current residence safe and comfortable?

1.Yes

2.No

**A21** What is the ownership of the house you live in?

1.Mine

2.Owned by children or relatives

3.Rent or help others take care of the house

**A22** Can you afford your own daily expenses?

1.Yes *Skip to A23*

2.No, receive funding

**A221** Where do you get the funding?

1.Family

2.Relatives or friends

3.Original company

4.Civil administration

5.Nursing home

**A23** How do you think your financial situation compares to your peers?

1.Better

2.Almost the same

3.Worse

**A24** Are you in charge of household expenses?

1.I am in charge of some major household expenses

2.I am in charge of almost all household expenses

3.I am in charge of some non-primary household expenses

4.I am only in charge of my own expenses

5.I am not in charge of any household expenses

**A25** Would you say your health is very good, good, fair, poor or very poor?

1.Very good

2.Good

3.Fair

4.Poor

5.Very poor

**A26** Have you been ill in the past two weeks?

1.Yes

2.No

**A27** Have you ever been diagnosed with chronic diseases by a doctor?

1.Yes

2.No *Skip to A28*

Have you been diagnosed with [conditions listed below, read one by one] by a doctor?

**A2701** Hypertension

**A2702** Diabetes

**A2703**  Heart disease

**A2704** Stroke or cerebrovascular disease

**A2705** Bronchitis, emphysema, asthma or pneumonia

**A2706** Tuberculosis

**A2707** Cataract

**A2708** Glaucoma

**A2709** Cancer

**A2710** Prostate disease

**A2711** Gastrointestinal ulcer

**A2712** Parkinson’s disease

**A2713** Bed sores

**A2714** Arthritis

**A2715** Dementia

**A2716** Epilepsy

**A2717**  Cholecystitis or cholelithiasis

**A2718** Dyslipidemia

**A2719** Rheumatism or Rheumatoid

**A2720** Chronic nephritis

**A2721** Breast hyperplasia

**A2722** Uterine fibroids

**A2723** Prostatic hyperplasia

**A2724** Hepatitis

**A2725** Other:____

**A272** Why did you diagnose that you have a chronic disease?(such as high blood pressure)

1.Feel unwell and see a doctor

2.See a doctor for other diseases

3.Routine physical examination

4.Community screening

5.See a doctor for advice from relatives and friends

6.See a doctor for publicity on TV, Intenet and other media

7.Other:____

**A273**  Are you receiving treatment, including non-drug therapy such as diet and exercise?

1.Yes

**AA273** What kind of treatment are you receiving:____

2.No

**A274** Where did you know the treatment?

1.Doctors

2.General practitioner

3.Physical examination

4.Intenet or media

5.Relatives and friends

6.Other:____

**A28** What kind of medical insurance do you take part in?

1.Government medical insurance

2.Urban employee medical insurance and Urban resident medical insurance

3.New cooperative medical insurance

4.Private medical Insurance

5.No insurance

**A29** What kind of pension insurance do you participate in?

1.Pension insurance for urban workers

2.Pension insurance for public institutions

3.Pension insurance for non-working urban residents

4.None of the above

**B CONCEPTION OF PROVIDING FOR THE AGED**

**B1** Who live with you? [Check as many as applied.]

1.Children *Skip B2*

2.Husband or wife

3.Other relatives

4.Friends

5.Live alone

6.Nursing home

**B2**  If you do not live with your child, how often do you contact your child?

1.Almost everyday

2.Once or twice a week

3.Once or twice a month

4.Once in a few months

5.Rarely contact

**B3** Which of the following modes of providing for the aged do you prefer?

1.Independent pension mode *Skip B4*

2.Family pension mode *Skip B4*

3.Community pension mode *Skip B4*

4.Institutional pension

**B4** Why do you choose to live in a nursing home?

1.I have children or they can’t take care of me

2.I don’t want to trouble my children

3.I don't have my own house, but I want to live separately from my children

4.I can communicate with other old people more

5.Other:____

**B5** Which of the following aspects do you think is more important in later life?

1.good health

2.Living environment

3.Food and clothing

4.Family company

5.Entertainment, study and life

6.Communicate with friends

7.Other:____

**B6** Which of the following contents of providing for the aged do you do you think is more important?

1.Financial help

2.Life care

3.Emotional support

4.Other:____

**B7** Who do you think should take more responsibility in providing for the aged?

1.Myself

2.Children

3.Government

4.Original company

**B8** Which of the following concepts of providing for the aged do you agree with?

1.I rely on the society (rely on social pension insurance, minimum living security system)

2.I rely on my children (spend money on children in middle age, and rely on children in old age)

3.I rely on myself (rely on my own savings, labor income, and pension)

**B9** Do you agree with the concept of “raising children for your old age”?

1.Strongly Agree

2.Quite agree

3.Fair

4.Don't agree

5.Totally disagree

**B10** Do you agree with “socialized mode of providing for the aged”?

1.I agree, communities and institutions can also provide good services

2.I disagree, only children can take good care of me

**B11** Do you think it's a shame to live in a nursing home?

1.Yes

2.No

**B12** Assuming that an elderly person has a spouse and adult children, and has a harmonious relationship with his children, what kind of living arrangement do you think is best for him?

1.Live with children

2.Do not live with his children, but live in the same village/community with his children

3.Do not live with children

4.Live in a nursing home

5.Other:____

**B13**  Assuming that an elderly person doesn’t have a spouse but has adult children, and has a harmonious relationship with his children, what kind of living arrangement do you think is best for him?

1.Live with children

2.Do not live with his children, but live in the same village/community with his children

3.Do not live with children

4.Live in a nursing home

5.Other:____

**C ASSESSMENT OF DEPENDENT PERSONALITY**

**C1** My daily life is full of things that keep me interested.

1.Yes

2.No

**C2** When I take a new job, I like to find out who it is important to be nice to.

1.Yes

2.No

**C3** At times I have very much wanted to leave home.

1.Yes

2.No

**C4** Evil spirits possess me at times.

1.Yes

2.No

**C5** I do not always tell the truth.

1.Yes

2.No

**C6** My feelings are not easily hurt.

1.Yes

2.No

**C7** I like poetry.

1.Yes

2.No

**C8** I do not mind being made fun of.

1.Yes

2.No

**C9** I do many things which I regret afterwards.

1.Yes

2.No

**C10** I like to go to parties and other affairs where there is lots of loud fun.

1.Yes

2.No

**C11** Some people are so bossy that I feel like doing the opposite of what they request even though I know they are right.

1.Yes

2.No

**C12** I have never done anything dangerous for the thrill of it.

1.Yes

2.No

**C13** My table manners are not quite as good at home as when I am out in company.

1.Yes

2.No

**C14** I believe I am being plotted against.

1.Yes

2.No

**C15** During the past few years I have been well most of the time.

1.Yes

2.No

**C16** It makes me uncomfortable to put on a stunt at a party even when others are doing the same sort of things.

1.Yes

2.No

**C17** I do not have a great fear of snakes.

1.Yes

2.No

**C18** My memory seems to be all right.

1.Yes

2.No

**C19** I am afraid of losing my mind.

1.Yes

2.No

**C20** There is something wrong with my mind.

1.Yes

2.No

**C21** I like to flirt.

1.Yes

2.No

**C22** In walking I am very careful to step over sidewalk cracks.

1.Yes

2.No

**C23** I very much like hunting.

1.Yes

2.No

**C24** I have at times stood in the way of people who were trying to do something, not because it amounted to much but because of the principle of the thing.

1.Yes

2.No

**C25** If I were an artist I would like to draw flowers.

1.Yes

2.No

**C26** I am entirely self-confident.

1.Yes

2.No

**C27** I am sure I am being talked about.

1.Yes

2.No

**C28** I have used alcohol excessively.

1.Yes

2.No

**C29** I have periods in which I feel unusually cheerful without any special reason.

1.Yes

2.No

**C30** Sometimes I become so excited that I find it hard to get to sleep.

1.Yes

2.No

**C31** I have certainly had more than my share of things to worry about.

1.Yes

2.No

**C32** I have no dread of going into a room by myself where other people have already gathered and are talking.

1.Yes

2.No

**C33** At parties I am more likely to sit by myself or with just one other person than to join in with the crowd.

1.Yes

2.No

**C34** People often disappoint me.

1.Yes

2.No

**C35** I enjoy children.

1.Yes

2.No

**C36** I am quite often not in on the gossip and talk of the group I belong to.

1.Yes

2.No

**C37** Sometime has been trying to influence my mind.

1.Yes

2.No

**C38** I can remember “playing sick” to get out of something.

1.Yes

2.No

**C39** I like parties and socials.

1.Yes

2.No

**C40** I am not easily angered.

1.Yes

2.No

**C41** I have done some bad things in the past that I never tell anybody about.

1.Yes

2.No

**C42** I like to keep people guessing what I’m going to do next.

1.Yes

2.No

**C43** I frequently ask people for advice.

1.Yes

2.No

**C44** The future is too uncertain for a person to make serious plans.

1.Yes

2.No

**C45** I am apt to take disappointments so keenly that I can’t put them out of my mind.

1.Yes

2.No

**C46** At times I think I am no good at all.

1.Yes

2.No

**C47** I usually work things out for myself rather than get someone to show me how.

1.Yes

2.No

**C48** I do not try to cover up my poor opinion or pity of people so that they won’t know how I feel.

1.Yes

2.No

**C49** It makes me angry to have people hurry me.

1.Yes

2.No

**C50** I have enjoyed using marijuana.

1.Yes

2.No

**C51** Mental illness is a sign of weakness.

1.Yes

2.No

**C52** I have a drug or alcohol problem.

1.Yes

2.No

**C53** Having to make important decisions makes me nervous.

1.Yes

2.No

**C54** I like making decisions and assigning jobs to others.

1.Yes

2.No

**C55**  When my life gets difficult, it makes me want to just give up.

1.Yes

2.No

**C56** I wish I could be as happy as others seem to be.

1.Yes

2.No

**C57** I work very long hours even though my job doesn’t require this.

1.Yes

2.No

**D SOCIAL RESOURCE CONDITIONS**

**D1** Who live with you? [Check as many as applied.]

1.No one

2.Husband or wife

3.Children

4.Grandchildren

5.Parents

6.Brothers and sisters

7.Other relatives (Does not include in-laws covered in the above categories)

8.Friends

9.Nonrelated paid help (includes free room)

10.Others

**D2** How many people do you know well enough to visit with in their homes?

1.Five or more

2.Three to four

3.One to two

4.None

**D3** About how many times did you talk to someone—friends, relatives, or others—on the telephone in the past week (either you called them or they called you)?

1.Once a day or more

2.2-6 times

3.Once

4.Not at all

**D4** How many times during the past week did you spend some time with someone who does not live with you; that is, you went to see them, or they came to see you, or you went out to do things together?

1.Once a day or more

2.2-6 times

3.Once

4.Not at all

**D5** Do you have someone you can trust and confide in?

1.Yes

2.No

**D6** Do you find yourself feeling lonely?

1.Quite often

2.Sometimes

3.Almost never

**D7** Do you see your relatives and friends as often as you want to or not?

1.As often as wants to

2.Not as often as wants to

**D8** Is there someone who would give you any help at all if you were sick or disabled?

1.Someone cares for indefinitely

2.Someone can provide short-term care (within six months)

3.Someone can help occasionally

4.None *Skip to E1*

**D9** Who provides the above services?

1.Husband or wife

2.Children

3.Grandchildren

4.Parents

5.Brothers and sisters

6.Other relatives (Does not include in-laws covered in the above categories)

7.Friends

8.Nonrelated paid help (includes free room)

9.Others

**E MEDICAL SERVICE CONDITIONS**

**E1** What is your choice of treatment after your illness?

1.Medical institution

2.Self treatment

3.No treatment

**E2** Why you have not been treated after your illness?

1.I have economic difficulties

2.I think the disease is not serious

3.It’s inconvenient to see a doctor

4.I’m busy with work and have no time

5.My family don't pay attention to it

6.Don't have medical insurance

7.Other

**E3** Which medical institution do you prefer when you are ill?

1.Second-class or above hospitals

2.Community health service organizations

3.Township health center

4.Village clinic

5.private clinic

6.Self-medication

**E4** How far is your home from the nearest medical institution?

1.＜2km

2.2.0-5.9km

3.6.0-9.9km

4.≥10.0km

**E5** How long does it take you to walk to the nearest medical institution?

1.＜10minutes

2.10-30minutes

3.31-60minutes

4.＞60minutes

**E6** Can you get timely treatment when you are seriously ill?

1.Yes

2.No

**E7** How many times have you been in hospital in the past six months?

1.Less than twice

2.2-3 times

3.4-6 times

4.More than 6 times

**E8** How many times have you been to the medical institution in the past six months?

1.Less than twice

2.2-3 times

3.4-6 times

4.More than 6 times

**E9** How much medical expenses have you borne(zi-fei) in the past six months?

1.＜2000 Yuan

2.2000-5999 Yuan

3.6000-10000 Yuan

4.＞10000 Yuan

**E10** Are you satisfied with the distribution of hospitals in your area?

1.Very satisfied

2.Quite satisfied

3.Fair

4.Not very satisfied

5.Dissatisfied

**E11** Are you satisfied with the attitude of the medical staff in the hospital?

1.Very satisfied

2.Quite satisfied

3.Fair

4.Not very satisfied

5.Dissatisfied

**E12** Are you satisfied with the waiting time?

1.Very satisfied

2.Quite satisfied

3.Fair

4.Not very satisfied

5.Dissatisfied

**F SERVICES AND FACILITIES AVAILABLE IN COMMUNITY**

**F1** In the past six months, how many times have you been to community health service organizations for treatment or received a general practitioner’s on-site service?

1.Less than twice

2.2-3 times

3.4-6 times

4.More than 6 times

**F2** What do you think of the medical skill of doctors in your community?

1.Very good

2.Good

3.Fair

4.Poor

5.Very poor

**F3** Are you satisfied with the attitude of the medical staff in your community?

1.Very satisfied

2.Quite satisfied

3.Fair

4.Not very satisfied

5.Dissatisfied

**F4** Whether the domestic garbage in the community is regularly transported by garbage trucks for disposal, and whether you are satisfied with the cleaning and disposal of the garbage?

1.Very satisfied

2.Quite satisfied

3.Fair

4.Not very satisfied

5.Dissatisfied

**F5** In the past six months, have there been any doctors or social workers who thoroughly checked and assessed your overall health?

1.Yes

2.No

**F6** Do you think it is necessary for someone to check and assess your overall health in this way?

1.Yes

2.No

**F7** Does your community provide the following services/facilities[listed below, read one by one]?

**F71** Family doctor / general practitioner contract service

**F72** Family beds

**F73**  Community geriatric ward

**F74** Day care services

**F75** Part-time worker

**F76** Home-based care services:

**F761** At-home haircut

**F762** Helping with bathing

**F763** Providing meals

**F764** Helping with going outside

**F765** Indoor cleaning

**F766** Helping with using the toilet

**F77** Rehabilitation equipment rental for the elderly

**F78** Transportation service

**F79**  Activity center for the elderly(chess and card room, reading room,chatting room etc.)

**F710** Volunteer Service

**F711**  Canteen for the elderly

**F712** Community nursing home

**F713** Short-term care homes for the elderly

**F714** Group apartment for the elderly with cognitive impairment

**F715** Counseling room, psychologist or psychological consultant

**F716** University for the elderly

**F717**  Emergency call or monitoring system

**F718** Are there any of the following places near your community?

**F7181** Cinema or theater

**F7182** Library

**F7183** Agricultural market and supermarket

**F7184** Cafe or tea room

**F719** Holding regular lectures on health knowledge

**F720** Establishing health records

**F721** First aid

**F722** Providing diet and medication guidance for the general elderly and chronic patients

**F723** Nursing care of severe chronic patients

**F724** Rehabilitation training and guidance

**F725** Providing guidance for caregivers

**F726** Regular follow-up visits for the elderly living alone or with disabilities by professional personnel

**F727** Skills training for self-care and prevention of autism

**F728** Reemployment assistance for the elderly

**F729** Organizing regular outdoor activities for the elderly

**F730** Screening for common diseases(e.g. regular blood pressure measurement)

**G THE MEASUREMENT OF SELF-EFFICACY**

**G1** You can always manage to solve difficult problems if you try hard enough.

1.Not at all true

2.Hardly true

3.Moderately true

4.Exactly true

**G2** If someone opposes you, you can find the means and ways to get what you want.

1.Not at all true

2.Hardly true

3.Moderately true

4.Exactly true

**G3** It is easy for you to stick to your aims and accomplish your goals.

1.Not at all true

2.Hardly true

3.Moderately true

4.Exactly true

**G4** You are confident that you could deal efficiently with unexpected events.

1.Not at all true

2.Hardly true

3.Moderately true

4.Exactly true

**G5** Thanks to your resourcefulness, you know how to handle unforeseen situations.

1.Not at all true

2.Hardly true

3.Moderately true

4.Exactly true

**G6** You can solve most problems if you invest the necessary effort.

1.Not at all true

2.Hardly true

3.Moderately true

4.Exactly true

**G7** You can remain calm when facing difficulties because you can rely on your coping abilities.

1.Not at all true

2.Hardly true

3.Moderately true

4.Exactly true

**G8** When you are confronted with a problem, you can usually find several solutions.

1.Not at all true

2.Hardly true

3.Moderately true

4.Exactly true

**G9** If you are in trouble, you can usually think of a solution.

1.Not at all true

2.Hardly true

3.Moderately true

4.Exactly true

**G10** You can usually handle whatever comes your way.

1.Not at all true

2.Hardly true

3.Moderately true

4.Exactly true

**H ASSESSMENT OF COGNITION AND PHYSICAL FUNCTION**

**HA** Do you feel that you forget a lot of things?

1.No

2.Yes, a little

3.Yes

4.Yes, a lot

**HB** Compared to last year, do you feel that you forget more things?

1.No

2.Yes, a little

3.Yes

4.Yes, a lot

**H1** Have you ever forgotten where you put things such as your wallet or keys?

1.Never

2.Sometimes

3.Frequently

4.Always

**H2** Have you ever forgotten a conversation that happened 5 minutes ago?

1.Never

2.Sometimes

3.Frequently

4.Always

**H3** Have you ever forgotten your own birth date?

1.Never

2.Sometimes

3.Frequently

4.Always

**H4** Have you ever forgotten what day and month it is?

1.Never

2.Sometimes

3.Frequently

4.Always

**H5** Have you ever forgotten where you are?

1.Never

2.Sometimes

3.Frequently

4.Always

**H6** Have you ever forgotten how to get back home?

1.Never

2.Sometimes

3.Frequently

4.Always

**H7** When the supply of electricity, gas or water ceases, can you deal appropriately with the issue?

1.Yes, without difficulty

2.Can most of the time

3.Can’t most of the time

4.Not at all

**H8** Can you make plans for the day?

1.Yes, without difficulty

2.Can most of the time

3.Can’t most of the time

4.Not at all

**H9** Can you select your own clothes appropriately according to the season or the situation?

1.Yes, without difficulty

2.Can most of the time

3.Can’t most of the time

4.Not at all

**H10** Can you buy things by yourself?

1.Yes, without difficulty

2.Can most of the time

3.Can’t most of the time

4.Not at all

**H11** Can you use the bus, the train or a car by yourself?

1.Yes, without difficulty

2.Can most of the time

3.Can’t most of the time

4.Not at all

**H12** Can you go to the bank to withdraw money or make a deposit, pay water, electricity, gas and other public use fees by yourself?

1.Yes, without difficulty

2.Can most of the time

3.Can’t most of the time

4.Not at all

**H13** Can you make phone calls?

1.Yes, without difficulty

2.Can most of the time

3.Can’t most of the time

4.Not at all

**H14** Can you prepare food by yourself?

1.Yes, without difficulty

2.Can most of the time

3.Can’t most of the time

4.Not at all

**H15** Can you take the correct quantity of medication at the right time of the day?

1.Yes, without difficulty

2.Can most of the time

3.Can’t most of the time

4.Not at all

**H16** Can you take a bath by yourself?

1.Yes, without difficulty

2.Need supervision or instructions

3.Need partial assistance

4.Need full assistance

**H17** Can you change clothes by yourself?

1.Yes, without difficulty

2.Need supervision or instructions

3.Need partial assistance

4.Need full assistance

**H18** Can you use the toilet by yourself?

1.Yes, without difficulty

2.Need supervision or instructions

3.Need partial assistance

4.Need full assistance

**H19** Can you take care of your own appearance?

1.Yes, without difficulty

2.Need supervision or instructions

3.Need partial assistance

4.Need full assistance

**H20** Can you eat on your own?

1.Yes, without difficulty

2.Need supervision or instructions

3.Need partial assistance

4.Need full assistance

**H21** Can you move around the house by yourself?

1.Yes, without difficulty

2.Need supervision or instructions

3.Need partial assistance

4.Need full assistance

**I GERIATRIC DEPREESION SCALE**

**I1** Are you basically satisfied with your life?

1.Yes

2.No

**I2** Have you dropped many of your activities and interests?

1.Yes

2.No

**I3** Do you feel that your life is empty?

1.Yes

2.No

**I4** Do you often feel bored?

1.Yes

2.No

**I5** Are you in good spirits most of the time?

1.Yes

2.No

**I6** Are you afraid that something bad is going to happen to you?

1.Yes

2.No

**I7** Do you feel happy most of the time?

1.Yes

2.No

**I8** Do you often feel helpless?

1.Yes

2.No

**I9** Do you prefer to stay at home rather than go out and do things?

1.Yes

2.No

**I10** Do you feel you have more problems with memory than most?

1.Yes

2.No

**I11** Do you think it is wonderful to be alive now?

1.Yes

2.No

**I12** Do you feel pretty worthless the way you are now?

1.Yes

2.No

**I13** Do you feel full of energy?

1.Yes

2.No

**I14** Do you feel that your situation is hopeless?

1.Yes

2.No

**I15** Do you think that most people are better off than you are?

1.Yes

2.No

**J MENTAL HEALTH**

**J1** What is your occupation or social activity before retirement? _____

**J2** Are you satisfied with the division of labor, remuneration, achievements, etc. of your occupation?

1.Very satisfied

2.Quite satisfied

3.Fair

4.Not very satisfied

5.Dissatisfied

**J3** Do you think living habits are very important to your health?

1.Very important

2.Quite important

3.Fair

4.Not very important

5.Unimportant

**J4** Do you attach great importance to your health and take active actions to improve your health?

1.Yes

2.No

**J5** Do you have a regular physical examination?

1.Yes **J50** How often do you have a physical examination?every_____years

2.No *Skip to J6*

**J6** Has anyone given you judgment and guidance on the lifestyle that should be improved based on the results of physical examination?

1.Yes

2.No

**J7** Which of the following ways do you get health knowledge?

1.Relatives and friends

2.Doctors

3.Posters

4.Books and newspapers

5.Brochures

6.Health lectures

7.TV Media

8.WeChat and other platforms

9.Other:_____

**J8** Have you ever suspected that you have suffered from mental disease, such as depression?

1.Yes

2.No

**J9** Have you ever been diagnosed with mental disease?

1.Yes

2.No

**J10** Have you ever had psychological counseling?

1.Yes

2.No

**J11** How many times have you had psychological counseling in the past six months?

1.Never

2.Once or twice

3.3-6 times

4.＞6 times

**J12** Where do you usually get mental health services, such as psychological counseling?

1.Never

2.Online

3.Community

4.Doctors

5.Relatives and friends

6.Other

**J13** Which of the following ways do you get mental health knowledge?

1.Relatives and friends

2.Doctors

3.Posters

4.Books and newspapers

5.Brochures

6.Health lectures

7.TV Media

8.WeChat and other platforms

9.Other

**J14**  If you suspect that you have or you do have a mental illness, what is the reason for not seeing a doctor?

1.Distrust of doctors

2.Stigma

3.Not knowing where to see a doctor

4.High cost

5.Other

**K GENETIC AND ENVIRONMENTAL HEALTH**

**K1** Have you ever had a genetic testing?

1.Yes

2.No

**K2** Have you been told or suspected that you have an inherited disease?

1.Yes

2.No

**K3** Have you received health education about inherited diseases?

1.Yes

2.No

**K4** Can you accept the cost of genetic testing? (6000 Yuan for 148 items)

1.Yes

2.No

**K5** What is the construction area of your current residence? _____m^2^

**K6** Does your residence have enough sunshine?

1.Yee

2.No

**K7** Does your residence have an indoor flushing toilet?

1.Yes

2.No

**K8** Does your residence have bath and shower equipment that can be used normally?

1.Yes

2.No

**K9** Is your residence well-ventilated?

1.Yes

2.No

**K10** Do you use heating facilities when the indoor temperature is below 15 degrees?

1.No

2.Using electrical heating

3.Using physical heating

4.Using solid fuel

5.Other:_____

**K11** Do you feel your residence is clean and comfortable?

1.Yes

2.No

**K12** Is there any fitness facility near your home?

1.Yes

2.No

**K13** Is your place of residence near a factory or toilet that is not clean?

1.Yes

2.No

**K14** Is there air pollution, noise, low air pressure, humidity and dryness in the surrounding environment of your residence for a long time?

1.Yes

2.No

**K15** Do you feel that the neighborhood of your residence is clean and comfortable?

1.Yes

2.No

**L THE COMPREHENSIVE ASSESSMENT OF PERSONALITY**

**L1** Does your mood often go up and down?

1.Yes

2.No

**L2** Would it upset you a lot to see a child or animal suffer?

1.Yes

2.No

**L3** Are you a talkative person?

1.Yes

2.No

**L4**  If you say you will do something do you always keep your promise no matter how inconvenient it might be?

1.Yes

2.No

**L5**  Do you ever feel “just miserable” for no reason?

1.Yes

2.No

**L6**  Would being in debt worry you?

1.Yes

2.No

**L7**  Are you rather lively?

1.Yes

2.No

**L8** Were you ever greedy by helping yourself to more than your share of anything?

1.Yes

2.No

**L9** Are you an irritable person?

1.Yes

2.No

**L10** Would you take drugs which may have strange or dangerous side effects?

1.Yes

2.No

**L11** Do you enjoy meeting new people?

1.Yes

2.No

**L12** Have you ever blamed someone for doing something you knew was really your fault?

1.Yes

2.No

**L13** Are your feelings easily hurt?

1.Yes

2.No

**L14** Have you ever insisted on having your own way?

1.Yes

2.No

**L15** Can you usually let yourself go and enjoy yourself at a lively party?

1.Yes

2.No

**L16** Are a ll your habits good and desirable ones?

1.Yes

2.No

**L17** Do you often feel “fed-up”?

1.Yes

2.No

**L18** Do good manners and cleanliness matter much to you?

1.Yes

2.No

**L19** Do you usually take the initiative in making new friends?

1.Yes

2.No

**L20** Have you ever said anything bad or nasty about anyone?

1.Yes

2.No

**L21** Do you often worry about things you should not have done or said?

1.Yes

2.No

**L22** Do you think marriage is old-fashioned and should be done away with?

1.Yes

2.No

**L23** Can you easily get some life into a rather dull party?

1.Yes

2.No

**L24** Have you ever taken anything that belonged to someone else?

1.Yes

2.No

**L25** Are you a worrier?

1.Yes

2.No

**L26** Do you like to cooperate with others?

1.Yes

2.No

**L27** Do you tend to keep in the background on social occasions?

1.Yes

2.No

**L28** Does it worry you if you know there are mistakes in your work?

1.Yes

2.No

**L29** Have you ever said anything bad or nasty about anyone?

1.Yes

2.No

**L30** Would you call yourself tense or “highly-strung” ?

1.Yes

2.No

**L31** Do you think people spend too much time safeguarding their future with savings and insurances?

1.Yes

2.No

**L32** Do you like mixing with people?

1.Yes

2.No

**L33** As a child did you ever talk back to your parents?

1.Yes

2.No

**L34** Do you worry too long after an embarrassing experience?

1.Yes

2.No

**L35** Do you try not to be rude to people?

1.Yes

2.No

**L36** Do you like plenty o f bustle and excitement around you?

1.Yes

2.No

**L37** Have you ever cheated at a game?

1.Yes

2.No

**L38** Do you suffer from “nervous” ?

1.Yes

2.No

**L39** Would you like other people to be afraid of you?

1.Yes

2.No

**L40** Have you ever taken advantage of someone?

1.Yes

2.No

**L41** Do you like telling jokes and funny stories to your friends?

1.Yes

2.No

**L42** Do you often feel lonely?

1.Yes

2.No

**L43** Do you think it’s better to follow social norms than to act in a personal way?

1.Yes

2.No

**L44** Do other people think o f you as being very lively?

1.Yes

2.No

**L45** Do you always practice what you preach?

1.Yes

2.No

**L46** Are you often troubled about feelings o f guilt?

1.Yes

2.No

**L47** Do you sometimes put o ff until tomorrow what you ought to do today?

1.Yes

2.No

**L48** Can you get a party going?

1.Yes

2.No

**HEIGHT** What's your height? _____cm

**WEIGHT** What's your weight? _____kg

**DBP** What was your diastolic blood pressure when you last measured your blood pressure? _____mmHg

**SBP** What was your systolic blood pressure when you last measured your blood pressure?_____mmHg
